# Supplementary material for: Accuracy and reproducibility of tumor size measurement using a deep-learning–based CDSS in resected lung cancer
Source: PLoS One. 2026 Mar 10;21(3):e0344445. doi: 10.1371/journal.pone.0344445 (PMC12974796; doi:10.1371/journal.pone.0344445)
Supplement: S1 Table — (DOCX) [file pone.0344445.s001.docx]

eTable 1. Technical Overview of MONCAD LCT (LuCAS-plus)

| **Component** | **Description** |
| --- | --- |
| Core Architecture | 3D Convolutional Neural Network (3D CNN) |
| Training Dataset | LIDC-IDRI (1,010 cases) |
| Annotation Protocol | Annotated independently by 4 thoracic radiologists; nodules with ≥3-agreement used |
| Training Volume | 800 CT scans with 1,620 nodules |
| Internal Validation | 88 CT scans with 185 nodules |
| Validation Performance | Sensitivity: 95%; False positives per scan: 0.78 |
| Key Functions | Nodule detection, density classification, segmentation, and diameter measurement |
| Segmentation Output | Full segmentation mask for each nodule |
| Size Measurement Method | Largest 2D diameter measured on axial plane |
| Part-Solid Nodule | Solid portion defined as regions with HU > –350 |
| Edge Case Handling | Additional rule-based post-processing applied |

|  |
| --- |

|  |
| --- |

|  |
| --- |

|  |  |
| --- | --- |
