# Supplementary material for: Accuracy and reproducibility of tumor size measurement using a deep-learning–based CDSS in resected lung cancer
Source: PLoS One. 2026 Mar 10;21(3):e0344445. doi: 10.1371/journal.pone.0344445 (PMC12974796; doi:10.1371/journal.pone.0344445)
Supplement: S2 Table — (DOCX) [file pone.0344445.s002.docx]

eTable 2. CT Scanner and Acquisition Parameters at Participating Institutions

| **Institution** | **Gachon University Gil Medical Center** | **Konyang University Hospital** | **Boramae Medical Center** |
| --- | --- | --- | --- |
| CT Scanner Model | Siemens  Somatom AS+, Somatom Definition Flash, Somatom **Edge, Somatom Force** | Siemens Somatom Definition Flash, **Somatom Force** | Philips Ingenuity CT |
| Detector Rows | **≥ 64** | **≥ 128** | 128 |
| kVp | 100~120 | 100~120 | 100~120 |
| Slice thickness | 1.25–3 mm for axial images | 1.25–3 mm for axial images | 1.0–1.25 mm for axial images |
| Slice interval | No interval | No interval | No interval |
| Reconstruction Kernel | Lung reconstruction kernel (B70f) | Lung reconstruction kernel | Lung reconstruction kernel |
| Contrast Use | Yes | Yes | Yes |
| Breath-Hold | Full inspiration | Full inspiration | Full inspiration |
