# Supplementary material for: Accuracy and reproducibility of tumor size measurement using a deep-learning–based CDSS in resected lung cancer
Source: PLoS One. 2026 Mar 10;21(3):e0344445. doi: 10.1371/journal.pone.0344445 (PMC12974796; doi:10.1371/journal.pone.0344445)
Supplement: Attachment — Submitted filename: Responses to Reviewers.docx [file pone.0344445.s004.docx]

18-January-2026

Dear Lorenzo Faggioni, M.D., Ph.D. Academic Editor, PLOS ONE

Thank you very much for the opportunity to revise our original article entitled “Accuracy and Reproducibility of Tumor Size Measurement Using a Deep-Learning–Based CDSS in Resected Lung Cancer (PONE-D-25- 39492).” After carefully reading the reviewer’ and editor’s comments, we have tried to improve the quality and legibility of the manuscript according to the points raised.

In the revised version, changes are indicated by highlighting. Individual points (E-editor’s comments and R-#1 = point 1 made by reviewer) are indicated in red.

PONE-D-25-39492

The diagnostic performance of deep-learning based clinical decision support system (CDSS) to predict tumor size for patients with resected lung cancer.

PLOS One

Dear Dr. Kim,

Thank you for submitting your manuscript to PLOS ONE. After careful consideration, we feel that it has merit but does not fully meet PLOS ONE’s publication criteria as it currently stands. Therefore, we invite you to submit a revised version of the manuscript that addresses the points raised during the review process.

Please submit your revised manuscript by Feb 05 2026 11:59PM. If you will need more time than this to complete your revisions, please reply to this message or contact the journal office at [plosone@plos.org](mailto:plosone@plos.org). When you're ready to submit your revision, log on to <https://www.editorialmanager.com/pone/> and select the 'Submissions Needing Revision' folder to locate your manuscript file.

Please include the following items when submitting your revised manuscript:

- A letter that responds to each point raised by the academic editor and reviewer(s). You should upload this letter as a separate file labeled 'Response to Reviewers'.
- A marked-up copy of your manuscript that highlights changes made to the original version. You should upload this as a separate file labeled 'Revised Manuscript with Track Changes'.
- An unmarked version of your revised paper without tracked changes. You should upload this as a separate file labeled 'Manuscript'.

If you would like to make changes to your financial disclosure, please include your updated statement in your cover letter. Guidelines for resubmitting your figure files are available below the reviewer comments at the end of this letter.

If applicable, we recommend that you deposit your laboratory protocols in [protocols.io](http://protocols.io/) to enhance the reproducibility of your results. Protocols.io assigns your protocol its own identifier (DOI) so that it can be cited independently in the future. For instructions see: [https://journals.plos.org/plosone/s/submission-guidelines#loc-laboratory-protocols](https://track.editorialmanager.com/CL0/https:%2F%2Fjournals.plos.org%2Fplosone%2Fs%2Fsubmission-guidelines%23loc-laboratory-protocols/1/010f019b49358371-f09d367e-6cd1-406d-afe0-703a8e4e0891-000000/pCQs54EZpFfcu9OcywHe9vBFZIseRH9NwnEW0Xgnxu8=242). Additionally, PLOS ONE offers an option for publishing peer-reviewed Lab Protocol articles, which describe protocols hosted on [protocols.io](http://protocols.io/). Read more information on sharing protocols at [https://plos.org/protocols?utm_medium=editorial-email&utm_source=authorletters&utm_campaign=protocols](https://track.editorialmanager.com/CL0/https:%2F%2Fplos.org%2Fprotocols%3Futm_medium=editorial-email%26utm_source=authorletters%26utm_campaign=protocols/1/010f019b49358371-f09d367e-6cd1-406d-afe0-703a8e4e0891-000000/ajjGmMNhWvnrcQ8V2KN5I8kpIS9p8oryPmlvKcMKzDw=242).

We look forward to receiving your revised manuscript.

Kind regards,

Lorenzo Faggioni, M.D., Ph.D.
Academic Editor
PLOS One

Journal Requirements:

When submitting your revision, we need you to address these additional requirements.

1. Please ensure that your manuscript meets PLOS ONE's style requirements, including those for file naming. The PLOS ONE style templates can be found at <https://journals.plos.org/plosone/s/file?id=wjVg/PLOSOne_formatting_sample_main_body.pdf> and <https://journals.plos.org/plosone/s/file?id=ba62/PLOSOne_formatting_sample_title_authors_affiliations.pdf>

🡪 Yes, we did.

2. PLOS requires an ORCID iD for the corresponding author in Editorial Manager on papers submitted after December 6th, 2016. Please ensure that you have an ORCID iD and that it is validated in Editorial Manager. To do this, go to ‘Update my Information’ (in the upper left-hand corner of the main menu), and click on the Fetch/Validate link next to the ORCID field. This will take you to the ORCID site and allow you to create a new iD or authenticate a pre-existing iD in Editorial Manager.

🡪 Yes, we did.

3. Please note that PLOS One has specific guidelines on code sharing for submissions in which author-generated code underpins the findings in the manuscript. In these cases, all author-generated code must be made available without restrictions upon publication of the work. Please review our guidelines at <https://journals.plos.org/plosone/s/materials-and-software-sharing#loc-sharing-code> and ensure that your code is shared in a way that follows best practice and facilitates reproducibility and reuse.

 🡪 Yes, we did.

4. We note that the grant information you provided in the ‘Funding Information’ and ‘Financial Disclosure’ sections do not match.

When you resubmit, please ensure that you provide the correct grant numbers for the awards you received for your study in the ‘Funding Information’ section.

 🡪 Yes, we did.

Financial disclosure

This research was supported by a grant of the Korean Health Technology R&D Project through the Korea Health Industry Development Institute (KHIDI), funded by the Ministry of Health & Welfare, Republic of Korea (grant number: RS-2022-KH129742). The funders had no role in study design, data collection and analysis, decision to publish, or preparation of the manuscript.

With Best Regards,

Jong-Yeup Kim

Department of Biomedical Informatics, Konyang University College of Medicine, Daejeon, Republic of Korea

jykim@kyuh.ac.kr

5. Thank you for stating the following in your Competing Interests section:
none

Please complete your Competing Interests on the online submission form to state any Competing Interests. If you have no competing interests, please state "The authors have declared that no competing interests exist.", as detailed online in our guide for authors at <http://journals.plos.org/plosone/s/submit-now>
This information should be included in your cover letter; we will change the online submission form on your behalf.

 🡪 Yes, we did.

The authors have declared that no competing interests exist.

6. Please update your submission to use the PLOS LaTeX template. The template and more information on our requirements for LaTeX submissions can be found at http://journals.plos.org/plosone/s/latex.

  🡪 Yes, we did.

7. We note that Figure(s) 1 and 2 in your submission contain copyrighted images. All PLOS content is published under the Creative Commons Attribution License (CC BY 4.0), which means that the manuscript, images, and Supporting Information files will be freely available online, and any third party is permitted to access, download, copy, distribute, and use these materials in any way, even commercially, with proper attribution. For more information, see our copyright guidelines: <http://journals.plos.org/plosone/s/licenses-and-copyright>.

We require you to either (1) present written permission from the copyright holder to publish these figures specifically under the CC BY 4.0 license, or (2) remove the figures from your submission:

a. You may seek permission from the original copyright holder of Figure(s) 1 and 2 to publish the content specifically under the CC BY 4.0 license.

We recommend that you contact the original copyright holder with the Content Permission Form (<http://journals.plos.org/plosone/s/file?id=7c09/content-permission-form.pdf>) and the following text:
“I request permission for the open-access journal PLOS ONE to publish XXX under the Creative Commons Attribution License (CCAL) CC BY 4.0 (<http://creativecommons.org/licenses/by/4.0/>). Please be aware that this license allows unrestricted use and distribution, even commercially, by third parties. Please reply and provide explicit written permission to publish XXX under a CC BY license and complete the attached form.”

Please upload the completed Content Permission Form or other proof of granted permissions as an "Other" file with your submission.

In the figure caption of the copyrighted figure, please include the following text: “Reprinted from [ref] under a CC BY license, with permission from [name of publisher], original copyright [original copyright year].”

 🡪 Thank you for raising this important point regarding Figures 1 and 2.
We confirm that these figures were created by one of the authors using anonymized CT images obtained from patients enrolled in this study.
The images were edited and annotated solely by the authors and do not contain any copyrighted material from external sources.
As the authors are the original creators and copyright holders of these figures, we confirm that we grant permission for publication under the CC BY 4.0 license.

b. If you are unable to obtain permission from the original copyright holder to publish these figures under the CC BY 4.0 license or if the copyright holder’s requirements are incompatible with the CC BY 4.0 license, please either i) remove the figure or ii) supply a replacement figure that complies with the CC BY 4.0 license. Please check copyright information on all replacement figures and update the figure caption with source information. If applicable, please specify in the figure caption text when a figure is similar but not identical to the original image and is therefore for illustrative purposes only.

8. [Please remove all personal information, ensure that the data shared are in accordance with participant consent, and re-upload a fully anonymized data set.

Note: spreadsheet columns with personal information must be removed and not hidden as all hidden columns will appear in the published file.

Additional guidance on preparing raw data for publication can be found in our Data Policy (https://journals.plos.org/plosone/s/data-availability#loc-human-research-participant-data-and-other-sensitive-data) and in the following article: http://www.bmj.com/content/340/bmj.c181.long.](http://track.editorialmanager.com/CL0/http:%2F%2Fwww.bmj.com%2Fcontent%2F340%2Fbmj.c181.long./1/010f019b49358371-f09d367e-6cd1-406d-afe0-703a8e4e0891-000000/tHqCVsGk49kPaLSW5Qm_4j5zasDzChdOeMrrZv94C7o=242)

 🡪 Yes, we did.

9. Please include captions for your Supporting Information files at the end of your manuscript, and update any in-text citations to match accordingly. Please see our Supporting Information guidelines for more information: http://journals.plos.org/plosone/s/supporting-information.

🡪 Yes, we did.

10. If the reviewer comments include a recommendation to cite specific previously published works, please review and evaluate these publications to determine whether they are relevant and should be cited. There is no requirement to cite these works unless the editor has indicated otherwise.

 🡪 Yes, we did.

[Note: HTML markup is below. Please do not edit.]

Reviewers' comments:

Reviewer's Responses to Questions

**Comments to the Author**

1. Is the manuscript technically sound, and do the data support the conclusions?

The manuscript must describe a technically sound piece of scientific research with data that supports the conclusions. Experiments must have been conducted rigorously, with appropriate controls, replication, and sample sizes. The conclusions must be drawn appropriately based on the data presented.

Reviewer #1: No

Reviewer #2: Yes

2. Has the statistical analysis been performed appropriately and rigorously?

Reviewer #1: Yes

Reviewer #2: Yes

3. Have the authors made all data underlying the findings in their manuscript fully available?

The [PLOS Data policy](http://track.editorialmanager.com/CL0/http:%2F%2Fwww.plosone.org%2Fstatic%2Fpolicies.action%23sharing/1/010f019b49358371-f09d367e-6cd1-406d-afe0-703a8e4e0891-000000/BSKlB0_bex54Be5ZMOdT1pjOA6vDOKHhXYvuVGe0Zmw=242) requires authors to make all data underlying the findings described in their manuscript fully available without restriction, with rare exception (please refer to the Data Availability Statement in the manuscript PDF file). The data should be provided as part of the manuscript or its supporting information, or deposited to a public repository. For example, in addition to summary statistics, the data points behind means, medians and variance measures should be available. If there are restrictions on publicly sharing data—e.g. participant privacy or use of data from a third party—those must be specified.

Reviewer #1: No

Reviewer #2: Yes

4. Is the manuscript presented in an intelligible fashion and written in standard English?

PLOS ONE does not copyedit accepted manuscripts, so the language in submitted articles must be clear, correct, and unambiguous. Any typographical or grammatical errors should be corrected at revision, so please note any specific errors here.

Reviewer #1: Yes

Reviewer #2: Yes

5. Review Comments to the Author

Please use the space provided to explain your answers to the questions above. You may also include additional comments for the author, including concerns about dual publication, research ethics, or publication ethics. (Please upload your review as an attachment if it exceeds 20,000 characters)

Reviewer #1: The study addresses an important clinical task; however, several methodological issues need clarification to ensure the soundness of the findings.

1. Title and aim: “predict” vs “measure”.
The manuscript repeatedly refers to “predicting tumour size” and “diagnostic performance”, but the study actually evaluates measurement accuracy and reproducibility of CT-based tumour size compared with pathology and radiologists. Since the reported ICCs show similar agreement between MONCAD LCT and routine reports, the emphasis on “prediction” seems conceptually misleading. I suggest reframing the aim toward measurement accuracy/consistency or defining more precisely what “predict” means in this context.

🡪We sincerely appreciate your thoughtful feedback. The manuscript has been revised to reflect your suggestion.

2. Description of the deep learning–based system.
The Methods section provides very limited information about MONCAD LCT despite describing it as a deep learning–based CDSS. Key functions such as nodule detection are not mentioned in Methods, although detection rates appear in Results. Likewise, the approach to delineating tumour margins, handling part-solid nodules, and determining the maximum solid-component diameter (2D plane vs 3D volumetric search) is not described. Some concise explanation of these components—within the limits of a commercial product—is necessary to understand how the system achieves its reported reproducibility.

🡪 Thank you for your insightful comment regarding the need to clarify the technical functions of the MONCAD LCT system. We have revised the <Methods section> and added a supplementary appendix (S1 table) to provide a more detailed description of the system’s architecture and core functionalities, including nodule detection, segmentation, and tumor size measurement.

3. Extraction and definition of radiology report measurements.
The process by which tumour size values were retrieved from routine radiology reports is insufficiently described, especially for part-solid nodules where both total and solid-component diameters may be documented. Without a clear extraction protocol, it is difficult to interpret whether the “report” measurements used for comparison reflect the original clinical assessment or choices made during retrospective data collection.

🡪 **Thank you for your insightful comment.**
We have clarified the process by which tumor size was extracted from the original radiology reports. Specifically, we now state that in part-solid nodules, the solid component’s maximum diameter was used, following the IASLC 8th edition TNM guidelines. We also confirm that no remeasurement was performed and all values reflect the original clinical interpretation. This clarification has been added to the Methods section of the revised manuscript

4. Use of the term CDSS.
Although MONCAD LCT may be marketed as a CDSS, in this study it supports only a narrow component of NSCLC evaluation (size assessment for T staging). It may be helpful to clarify this limited scope or use a more precise term.

Clarifying these points would significantly strengthen the methodological transparency and interpretation of the work.

**🡪Thank you for pointing out the need to clarify the scope of the CDSS function in our study.**
We agree that, although MONCAD LCT is marketed as a CDSS, in this study it was applied solely for its tumor size measurement function on preoperative CT, relevant to T-staging in NSCLC. To avoid confusion, we have clarified this point in the revised manuscript by specifying the limited scope of its use in our context at <Discussion section>. This clarification enhances the transparency and accuracy of the methodological description.

Reviewer #2: Well written and interesting. The protocol for acquiring the analysed CT scans is missing, which is known to be one of the factors that can influence the automatic analyses performed by the software. Can you provide?

🡪 Thank you for your helpful comment. We agree that CT acquisition protocols may influence the performance of AI-based image analysis. We have added a description of the CT acquisition methods in the **Methods and S2 table.**
All scans were obtained at three institutions using multi-detector CT scanners with ≥64 detector rows and standard lung imaging protocols. Images were reconstructed with thin slices (1.0–3.0 mm) using lung kernels, and most scans were acquired with contrast at full inspiration in the supine position. These parameters align with clinical practice guidelines for lung cancer evaluation and provide a consistent basis for automated analysis by the CDSS.

We have also included a supplementary table (Supplementary Table X) summarizing the CT scanner models and acquisition parameters used at the three participating institutions. This provides further transparency and supports the reproducibility and generalizability of the automated analysis.

6. PLOS authors have the option to publish the peer review history of their article ([what does this mean?](https://track.editorialmanager.com/CL0/https:%2F%2Fjournals.plos.org%2Fplosone%2Fs%2Feditorial-and-peer-review-process%23loc-peer-review-history/1/010f019b49358371-f09d367e-6cd1-406d-afe0-703a8e4e0891-000000/BQlXq0ZtnOROXivd316csrJm3AxXuwzUTkXqSYmaZu0=242)). If published, this will include your full peer review and any attached files.


If you choose “no”, your identity will remain anonymous but your review may still be made public.


**Do you want your identity to be public for this peer review?** For information about this choice, including consent withdrawal, please see our [Privacy Policy](https://track.editorialmanager.com/CL0/https:%2F%2Fwww.plos.org%2Fprivacy-policy/1/010f019b49358371-f09d367e-6cd1-406d-afe0-703a8e4e0891-000000/VzflBxci5Z8goxy7CF1GQowY6-8z6ePUN5q7fgogDPw=242).

Reviewer #1: No

Reviewer #2: No

[NOTE: If reviewer comments were submitted as an attachment file, they will be attached to this email and accessible via the submission site. Please log into your account, locate the manuscript record, and check for the action link "View Attachments". If this link does not appear, there are no attachment files.]

To ensure your figures meet our technical requirements, please review our figure guidelines: <https://journals.plos.org/plosone/s/figures>

You may also use PLOS’s free figure tool, NAAS, to help you prepare publication quality figures: <https://journals.plos.org/plosone/s/figures#loc-tools-for-figure-preparation>.

NAAS will assess whether your figures meet our technical requirements by comparing each figure against our figure specifications.

Thank you again for giving us great honor to resubmit our manuscript to this prestigious journal. We have done our best to respond to all points indicated by the reviewers. We hope you find the revised manuscript acceptable for publication in PLOS ONE.

The authors confirm the manuscript has not been published previously and that it will not be submitted for publication elsewhere and the authors have declared that no competing interests exist.

Uploaded:

-Revised and highlighted versions of the revised manuscript (‘Manuscript’ file and

‘Revised Manuscript with Track Changes’ file)

-Rebuttal letter (‘Responses to Reviewers’ file)

With Best Regards,

[Jong-Yeup Kim](https://pubmed.ncbi.nlm.nih.gov/?sort=date&term=Kim+JY&cauthor_id=39666629)

Department of Biomedical Informatics, Konyang University College of Medicine, Daejeon, Republic of Korea

[jykim@kyuh.ac.kr](mailto:jykim@kyuh.ac.kr)
